# Cardiometabolic Index as a Mediator in the Association Between Estimated Glucose Disposal Rate and Depressive Symptoms: A Population‐Based Study

**DOI:** 10.1002/brb3.71247

**Published:** 2026-02-05

**Authors:** Mimi Li, Shujuan Wu, Xiaofang Ye, Binbin Yu, Wanli Huang, Lichao Ye, Chunnuan Chen

**Affiliations:** ^1^ Department of Neurology The Second Affiliated Hospital of Fujian Medical University Quanzhou Fujian China

**Keywords:** cardiometabolic index, cross‐sectional study, depression, estimated glucose disposal rate

## Abstract

**Background:**

Estimated glucose disposal rate (eGDR) emerged as an innovative marker for insulin resistance, and this study was designed to investigate the connection between eGDR and depressive symptoms.

**Methods:**

Information from the National Health and Nutrition Examination Survey (NHANES) was processed within the cross‐sectional research. Relationships between depressive symptoms and eGDR were examined using weighted logistic regression models, restricted cubic splines (RCS), sensitivity analyses, and subgroup comparisons. Receiver operating characteristic (ROC) curve analysis assessed the capacity for prediction of eGDR, relative to triglyceride‐glucose (TyG) index, Homeostasis Model Assessment of Insulin Resistance (HOMA‐IR), and glycated hemoglobin (HbA1c). Mediation analysis was also applied to assess cardiometabolic index (CMI)’s potential role in the eGDR‐depression relationship.

**Results:**

This analysis comprised 12,191 individuals stratified by eGDR tertiles (T1: <6.15; T2: 6.15–9.44; T3: ≥9.44 mg/kg/min). Multivariate logistic regression, adjusted for covariates, found that T3 had a significantly lower odds ratio for depressive symptoms than T1 (OR = 0.68, 95% CI: 0.48–0.97). RCS curves confirmed a linear trend (P for non‐linearity = 0.764). The negative association was particularly evident in participants under 60, non‐Hispanic Black individuals, those living alone, and those without cardiovascular disease. ROC analysis indicated that eGDR had better discriminative power for depressive symptoms than TyG, HOMA‐IR, and HbA1c (*p* < 0.05). Additionally, CMI mediated approximately 11.2% (95% CI: 4.2%–32.7%) of the total effect of eGDR on depressive symptoms.

**Conclusions:**

Higher eGDR correlates with lower likelihood of depressive symptoms, with CMI acting as a mediator. Reducing insulin resistance and monitoring CMI could help decrease the occurrence of depression.

## Graphical abstract

This cross‐sectional study included individuals who took part in the NHANES from 2003 to 2018. Our results demonstrated that higher eGDR correlates with lower likelihood of depressive symptoms, with CMI acting as a mediator. Reducing insulin resistance and monitoring CMI could help decrease the occurrence of depression.

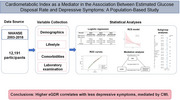

**Abbreviations**
BMI: Body mass index
CMI: Cardiometabolic index
CVD: Cardiovascular disease
DM: Diabetes mellitus
eGDR: Estimated glucose disposal rate
HbA1c: Glycated hemoglobin
HDL‐C: High‐density lipoprotein cholesterol
HOMA‐IR: Homeostasis Model Assessment of Insulin Resistance
HT: hypertension
IR: insulin resistance
LDL‐C: Low‐density lipoprotein cholesterol
NHANES: National Health and Nutrition Examination Survey
PHQ‐9: Patient Health Questionnaire‐9
PIR: Family poverty income ratio
RCS: Regression analysis, restricted cubic spline
ROC: Receiver operating characteristic
T2DM: Type 2 Diabetes Mellitus
TC: Total cholesterol
TG: Triglyceride
TyG: triglyceride‐glucose
WHtR: Waist‐to‐height ratio

## 1 Introduction

Depression, marked by persistent and severe low mood, is widespread mental disorder that significantly impacts life quality, and elevating medical comorbidities and mortality risks (O'Connor et al. 2023). As defined by the Anxiety and Depression Association of America, depression represents a prolonged emotional state (extending beyond two weeks) marked by feelings of despair, lack of motivation, pervasive sadness, and withdrawal from life's activities, with substantial interference in routine tasks (Sforzini et al. 2022). Global prevalence of depression has surged dramatically, with approximately 280 million cases currently identified (GBD 2019 Mental Disorders Collaborators 2022). Numerous population‐based observational research have emphasized the association between diabetes and depression, particularly as a result of insulin resistance (IR) (Cui et al. 2022). Although recognized as the reference method for assessing IR, the hyperinsulinemic‐euglycemic clamp faces significant limitations in clinical and population‐based research due to its invasive procedure, technical complexity, and substantial resource requirements (Xiao et al. 2024). Consequently, alternative indices including triglyceride‐glucose (TyG) index (Vasques et al. 2011), Homeostasis Model Assessment of Insulin Resistance (HOMA‐IR) (Matthews et al. 1985), and estimated glucose disposal rate (eGDR) (Orchard et al. 2003), which provide more efficient alternatives for insulin resistance assessment in routine practice.

As a clinically accessible tool for assessing IR in diabetic patients, the eGDR incorporates glycated hemoglobin A (HbA1c), waist circumference, and hypertension (HT) to provide a simple and accurate alternative to the euglycemic hyperinsulinemic clamp (Nyström et al. 2018). Due to its convenient nature, eGDR is well‐suited for extensive population‐based research, offering a practical approach to assessing IR across various patient populations (Zabala et al. 2021). While longitudinal data confirm TyG's predictive value for depression in older adults in a longitudinal study of 19,114 participants (Forbes et al. 2025), the biological plausibility of eGDR as a multisystemic metric may offer superior mechanistic insights. Unlike TyG and MetS‐IR, the eGDR incorporates stable clinical parameters—including blood pressure and HbA1c—to provide a comprehensive assessment of metabolic health in diabetes. This multidimensional approach may enhance its clinical feasibility for routine measurement in diabetic patients and populations at high risk for diabetes. Moreover, this metric proves valuable for individuals with and without type 2 diabetes. Its robust connections to all‐cause mortality, cardiovascular‐caused deaths, and diabetes complications position it as a promising indicator for predicting various negative health consequences (Meng et al. 2023; Gudenkauf et al. 2024). The relation between eGDR and depressive symptoms remains uncertain.

Multiple investigations have implicated lipid metabolism abnormalities in depression pathogenesis and clinical progression (Kroenke et al. 2001; Rafiee et al. 2024). As a recently developed indicator, cardiometabolic index (CMI) provides a composite assessment of visceral obesity and circulating lipid concentrations (Larsson et al. 2023; Song et al. 2025). Meanwhile, eGDR and CMI are closely associated with metabolic dysregulation. More recently, epidemiological researches stated the independent relation between CMI values and depression occurrence (Zhou et al. 2024). Of particular mechanistic interest, previous work by Shao et al. evaluated the association between eGDR and depression in the general population using neutrophils and leukocytes as mediators, though the mediation effects were relatively modest (4.0% and 3.6%, respectively) (Shao et al. 2025). Another subsequent analysis in diabetic subpopulations by Chen et al. further confirmed this relationship but similarly identified a limited mediation effect of the atherogenic index of plasma as 9.6% (Chen et al. 2024). Critically, these findings point to an important knowledge gap: while the eGDR‐depression association is established, the predominant biological mediators remain poorly characterized, particularly regarding lipid‐metabolic pathways.

Therefore, we investigated the relationships between eGDR and depressive symptoms using NHANES data to reveal scientific evidence to support the screening of high‐risk populations for depression. Furthermore, we postulated that eGDR might correlate with CMI and that CMI could potentially mediate the relation between eGDR and depression. Meanwhile, we conducted mediation analysis to investigate this potential mediation pathway.

## 2 Methods

### 2.1 Data Source and Participants

**FIGURE 1:**
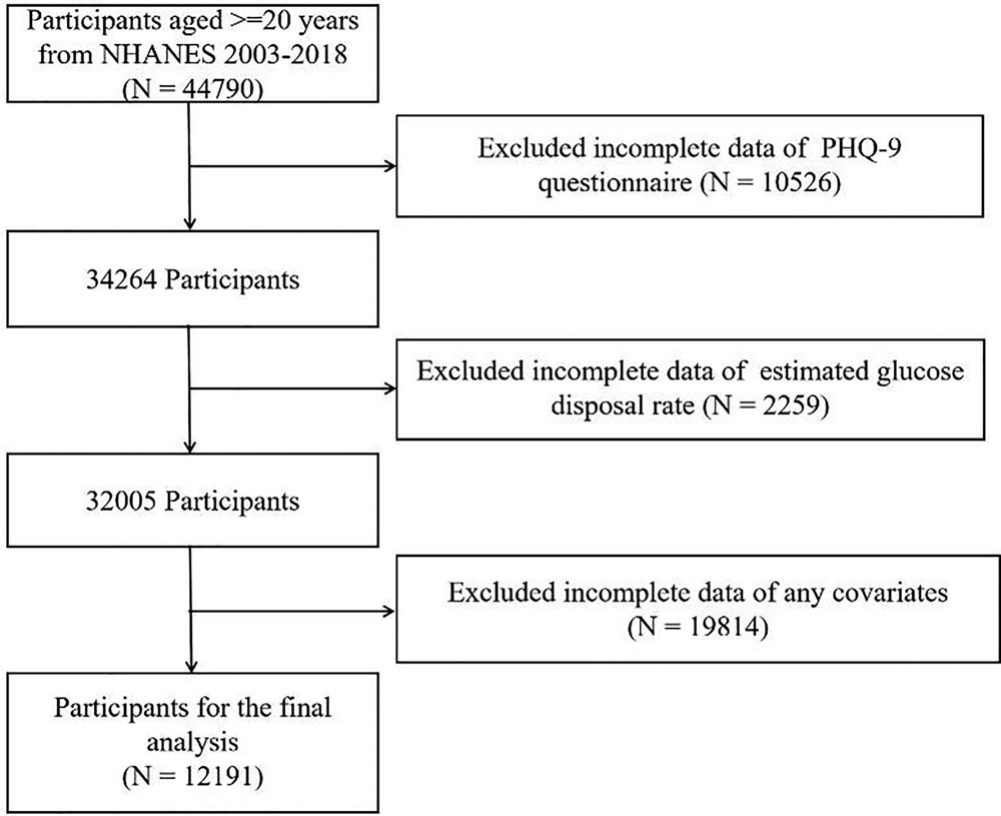
Flow diagram of study cohort selection.

Th national Health and Nutrition Examination Survey (NHANES) is aimed at enhancing the understanding of dietary and health patterns of residents across the United States. We utilized data from 2003 to 2018, in which written informed consent was sourced from all subjects, and detailed ethical information can be found at https://www.cdc.gov/nchs/nhanes/index.htm. All subjects were excluded referring to the criteria below: data absent for Patient Health Questionnaire‐9 (PHQ‐9), eGDR, covariates, or age under 20 years. The final analytical sample consisted of 12,191 eligible participants. A detailed flowchart outlining the selection criteria, including inclusion and exclusion steps, is presented in Figure 1.

### 2.2 Variables

#### 2.2.1 Depressive Symptoms

This investigation utilized the PHQ‐9 to assess depressive symptom severity during the preceding two‐week period. The instrument's nine items were scored on a 4‐point Likert scale, spanning 0 (“not at all”) to 3 (“nearly every day”). Total scores were derived by aggregating responses across all assessment items, with a threshold of ≥10 established as the cutoff for clinically significant depressive symptoms (Watson et al. 2021).

#### 2.2.2 The eGDR and Other IR Indicators

eGDR was computed from the following formula, as previously reported: eGDR (mg/kg/min) = 21.158−(0.09×waist circumference [cm]) − (3.407×hypertension) − 0.551×HbA1C (%) (Nyström et al. 2018). Hypertension diagnosis was based on either: (1) three consecutive blood pressure readings showing systolic values ≥140 mmHg and/or diastolic values ≥90 mmHg, or (2) documented self‐reported history of hypertension. Study population was stratified into three groups based on eGDR levels (T1<6.15 mg/kg/min; 6.15 mg/kg/min≤T2<9.44 mg/kg/min; T3≥9.44 mg/kg/min), with T1 serving as the reference group. TyG was computed from formula below: TyG = Ln [fasting blood glucose (mg/dL) × triglycerides (mg/dL)/2] (Penno et al. 2021). HOMA‐IR was computed through the equation below: HOMA‐IR = fasting insulin (mU/mL) × fasting plasma glucose (mg/dL)/405 (Garofolo et al. 2020).

#### 2.2.3 Ascertainment of Cardiometabolic Index (CMI)

Waist‐to‐height ratio (WHtR) was derived by taking the quotient of waist circumference (cm) and standing height (cm). CMI was then computed through the established formula, as previously reported: CMI = TG (mmol/L)/HDL‐C (mmol/L) × WHtR (Song et al. 2025).

#### 2.2.4 Covariates

Demographic and socioeconomic factors considered in the analyses comprised marital status, race, educational attainment, age, the family poverty income ratio (PIR), and sex (Rauscher et al. 2024), and smoking and alcohol consumption. Moreover, physical examination relevant factors consisted of body mass index (BMI) and waist circumference. Comorbidities comprised cardiovascular disease (CVD), hypertension, and diabetes mellitus (DM). Laboratory tests results included measurements of serum creatinine, high‐density lipoprotein cholesterol (HDL‐C), uric acid, triglyceride (TG), total cholesterol (TC), low‐density lipoprotein cholesterol (LDL‐C). Detailed information could be achieved within supplementary information (Table S1).

### 2.3 Statistical Analyses

Unless otherwise specified, values are presented as either the number of participants (weighted percentages) or the weighted means (standardized errors). For continuous data, differences among the three cohorts were assessed by both Kruskal‐Wallis H test and one‐way ANOVA. For categorical variables, chi‐square tests were employed. Trough weighted models of logistic regression, relation among eGDR, eGDR tertiles, and depressive symptom was analyzed. Model 1 remained crude. Model 2 controlled for educational attainment, age, marital status, race, sex. Model 3 further controls for PIR, alcohol consumption, smoking status, BMI, cardiovascular disease, diabetes mellitus, and total cholesterol. The association between depressive symptoms and eGDR was examined by weighted restricted cubic splines, with 3 knots located at the 25^th^, 50^th^, and 75^th^ percentiles selected through minimization of the Akaike information criterion. Sensitivity analyses excluding adjustments for diabetes mellitus and total cholesterol were conducted to verify findings. Subgroup evaluations were performed stratified by body mass index, smoking behavior, age, marital status, PIR, cardiovascular disease, race, education level, diabetes mellitus status, and sex. To compare the predictive performance of different glycemic markers, we conducted receiver operating characteristic (ROC) analyses to assess how well eGDR, the TyG index, HOMA‐IR, and HbA1c could identify individuals with depressive symptoms. Furthermore, we employed a four‐way decomposition approach to reveal mediating effects of CMI on depressive symptoms using the CMAverse R package (Nyström et al. 2017). Statistical analyses used R 4.3.1, with *P* < 0.05 considered statistically significant.

## 3 Results

### 3.1 Baseline Characteristics

The final analysis comprised 12,191 eligible subjects. Participants were stratified into tertiles according to eGDR tertiles (Table 1). Significant disparities in clinical features were observed across these groups. Individuals in the highest eGDR group showed distinct characteristics compared to the lowest tertile: younger age, greater proportion of females, lower percentage of non‐Hispanic Whites, higher education levels, and increased PIR. They also had reduced smoking prevalence and lower rates of depressive symptoms, HTN, DM, and CVD. Laboratory parameters including HbA1c, HDL‐C, LDL‐C, FBG, TG, and TC were all significantly lower. Moreover, participants with higher eGDR demonstrated reduced levels of BMI, WC, TyG, HOMA‐IR and CMI (*p* < 0.001).

**TABLE 1:** Population information weighted by eGDR tertiles^a^.

Characteristics	Total	T1	T2	T3	*p*‐value
**Number**	12,191	4,064	4,064	4,063	
**Age (years)**	47.02 (0.28)	56.08 (0.34)	48.05 (0.35)	38.95 (0.36)	< 0.001
**Sex (%)**					< 0.001
Male	6,076 (49.28)	2,178 (54.19)	2,105 (52.34)	1,793 (42.62)	
Female	6,115 (50.72)	1,886 (45.81)	1,959 (47.66)	2,270 (57.38)	
**Race (%)**					
Non‐Hispanic White	5,534 (69.68)	1,906 (71.51)	1,839 (69.65)	1,789 (68.28)	0.036
Non‐Hispanic Black	2,374 (10.47)	1,026 (13.51)	706 (9.35)	642 (9.09)	< 0.001
Mexican American	1,873 (7.94)	530 (6.25)	734 (9.40)	609 (7.96)	< 0.001
Other Race	2,410 (11.90)	602 (8.73)	785 (11.61)	1,023 (14.67)	< 0.001
**Educational Attainment (%)**					< 0.001
High school or less	5,574 (38.06)	2,102 (44.58)	1,935 (39.92)	1,537 (31.24)	
More than high school	6,617 (61.94)	1,962 (55.42)	2,129 (60.08)	2,526 (68.76)	
**Marital Status (%)**					< 0.001
Married or living with partner	7,447 (64.48)	2,480 (65.61)	2,594 (67.37)	2,373 (60.97)	
Living alone	4,744 (35.52)	1,584 (34.39)	1,470 (32.63)	1,690 (39.03)	
**PIR (%)**					
Low	2,423 (13.33)	819 (13.01)	792 (13.19)	812 (13.70)	0.681
Middle	6,518 (49.68)	2,275 (52.92)	2,219 (50.69)	2,024 (46.21)	< 0.001
High	3,250 (36.99)	970 (34.06)	1,053 (36.11)	1,227 (40.09)	< 0.001
**Smoking status (%)**					
Never	6,684 (54.69)	2,033 (49.70)	2,150 (53.03)	2,501 (60.14)	< 0.001
Now	2,454 (19.94)	700 (16.65)	899 (21.86)	855 (20.80)	< 0.001
Former	3,053 (25.36)	1,331 (33.65)	1,015 (25.12)	707 (19.07)	< 0.001
**Drinking status (%)**					
Never	1,603 (10.26)	567 (11.48)	528 (9.81)	508 (9.70)	0.060
Mild	4,299 (37.87)	1,443 (39.26)	1,426 (37.29)	1,430 (37.31)	0.258
Moderate	1,874 (17.53)	504 (14.85)	616 (16.52)	754 (20.56)	< 0.001
Heavy	2,469 (21.40)	640 (15.90)	854 (22.72)	975 (24.54)	< 0.001
Former	1946 (12.94)	910 (18.51)	640 (13.66)	396 (7.89)	< 0.001
**Hypertension (%)**					< 0.001
No	7,095 (63.03)	254 (6.74)	2,778 (71.13)	4,063 (100.00)	
Yes	5,096 (36.97)	3,810 (93.26)	1,286 (28.87)	0 (0.00)	
**DM (%)**					< 0.001
No	9,721 (85.01)	2,343 (63.75)	3,441 (89.13)	3,937 (98.01)	
Yes	2,470 (14.99)	1,721 (36.25)	623 (10.87)	126 (1.99)	
**CVD (%)**					< 0.001
No	10,896 (91.47)	3,216 (81.72)	3,710 (92.57)	3,970 (98.16)	
Yes	1,295 (8.53)	848 (18.28)	354 (7.43)	93 (1.84)	
**Depressive symptoms (%)**					< 0.001
No	11,195 (92.94)	3,633 (90.73)	3,746 (93.02)	3,816 (94.62)	
Yes	996 (7.06)	431 (9.27)	318 (6.98)	247 (5.38)	
**FBG (mg/dL)**	105.42 (0.38)	121.01 (0.95)	103.09 (0.35)	95.28 (0.23)	< 0.001
**TC (mg/dL)**	192.22 (0.57)	191.98 (0.97)	197.08 (0.98)	187.98 (0.77)	< 0.001
**TG (mg/dL)**	117.09 (0.98)	140.88 (1.84)	123.33 (1.59)	92.67 (1.05)	< 0.001
**HDL‐C (mg/dL)**	54.54 (0.24)	49.73 (0.31)	53.19 (0.40)	59.54 (0.35)	< 0.001
**LDL‐C (mg/dL)**	114.26 (0.46)	114.07 (0.80)	119.22 (0.74)	109.91 (0.67)	< 0.001
**HbA1c (%)**	5.59 (0.01)	6.11 (0.03)	5.52 (0.01)	5.24 (0.01)	< 0.001
**BMI (kg/m^2^)**	28.95 (0.10)	34.20 (0.18)	29.77 (0.11)	24.08 (0.06)	< 0.001
**WC (cm)**	99.11 (0.27)	113.71 (0.35)	101.89 (0.28)	85.09 (0.16)	< 0.001
**HOMA‐IR**	3.43 (0.06)	5.72 (0.16)	3.26 (0.05)	1.77 (0.03)	< 0.001
**TyG**	8.56 (0.01)	8.88 (0.02)	8.61 (0.01)	8.25 (0.01)	< 0.001
**CMI**	0.66(0.01)	0.95(0.02)	0.71(0.01)	0.40(0.01)	< 0.001

Abbreviations: BMI, body mass index; CMI, cardiometabolic index; CVD, cardiovascular disease; DM, diabetes mellitus; eGDR, estimated glucose disposal rate; FBG, fasting blood glucose; HbA1c, glycosylated hemoglobin A1C; HDL‐C, high‐density lipoprotein cholesterol; HOMA‐IR, homeostatic model assessment for insulin resistance; LDL‐C, low‐density lipoprotein cholesterol; PIR, family poverty income ratio; T, tertiles; TC, total cholesterol; TG, triglyceride; TyG, triglyceride and glucose index; WC, waist circumference.

^a^ Values are weighted means (standardized errors) or number of participants (weighted percentages) unless otherwise indicated.

### 3.2 Relation Between Depressive Symptom and eGDR

The potential relation between depressive symptoms and eGDR was examined using three models (Table 2). Analysis revealed an inverse association of eGDR with depressive symptoms that reached statistical significance. Model 3 indicated that each 1‐unit increase in eGDR corresponded to a significantly lower likelihood of experiencing depressive symptoms (OR = 0.93, 95% CI: 0.87‐0.99, *p* = 0.035). In the full Model 3, in addition to eGDR, gender, PIR, marital status, smoking status, and CVD were also associated with depression (Table S2).

**TABLE 2:** Relation between depressive symptom and eGDR evaluated by weighted logistic regression with multiple covariates^a^.

Characteristic	Model 1 OR (95%CI), *p*‐value	Model 2 OR (95%CI), *p*‐value	Model 3 OR (95%CI), *p*‐value
eGDR (continuous)	0.91 (0.88, 0.94), < 0.001	0.89 (0.86, 0.92), < 0.001	0.93 (0.87, 0.99), 0.035
eGDR (categorical)			
T1	Reference	Reference	Reference
T2	0.73 (0.60, 0.89), 0.002	0.70 (0.57, 0.86), < 0.001	0.81 (0.64, 1.02), 0.073
T3	0.56 (0.45, 0.69), <0.001	0.47 (0.37, 0.60), < 0.001	0.68 (0.48, 0.97), 0.031
P for trend	< 0.001	< 0.001	0.029

Abbreviations: eGDR, estimated glucose disposal rate; T, tertiles.

^a^Model 1: unadjusted; Model 2: adjusted for age, sex, race, educational attainment, and marital status; Model 3: adjusted for age, sex, race, education attainment, marital status, BMI, PIR, smoking status, drinking status, CVD, DM, and TC.

For sensitivity analysis, we converted continuous eGDR values into tertiles (low, medium, high) to assess potential dose‐response relationships. In comparison to the participants in T1 group, ORs for depressive symptom prevalence in the T2 and T3 groups were 0.70 (95% CI: 0.57–0.86) and 0.47 (95% CI: 0.37–0.60), respectively, for Model 2.

Using T1 as the reference group, no significant difference was found between T1 and T2 in Model 3 (OR = 0.81, 95% CI: 0.64‐1.02). However, compared to T1, T3 presented a notable negative relation with depressive symptoms (OR = 0.68, 95% CI: 0.48‐0.97).

**FIGURE 2:**
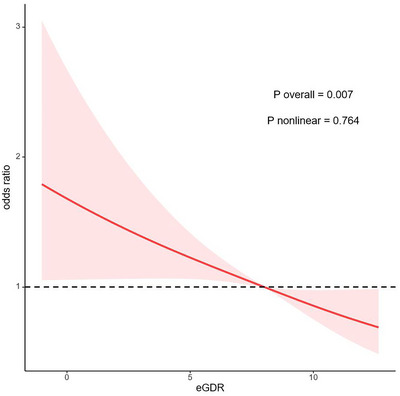
Relation between depressive symptom and eGDR. Adjustment factors included smoking habit, BMI, marital status, age, drinking status, DM, education attainment, race, sex, CVD, PIR, and TC.

Weighted multivariate RCS modeling assessed potential nonlinear eGDR‐depression relations (Figure 2). The 0.764 nonlinearity *p*‐value supported a linear association (overall *P* < 0.001). Furthermore, increasing eGDR levels correlated with progressively lower PHQ‐9 scores (Figure S1).

### 3.3 Subgroup Analysis

**FIGURE 3:**
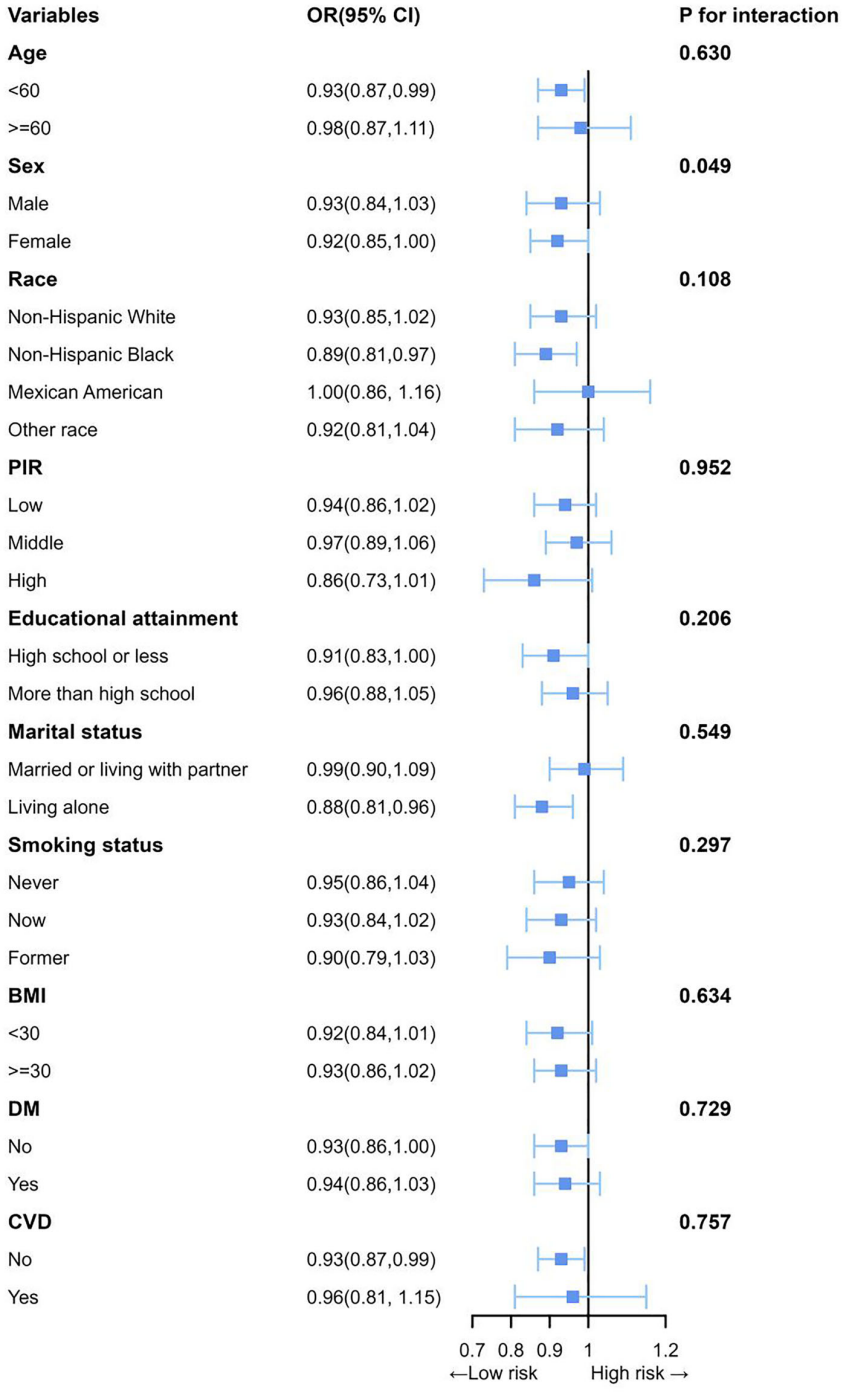
Subgroup analyses between eGDR and depressive symptoms. Each subgroup analysis controlled factors comprising CVD, drinking status, BMI, race, TC, education attainment, smoking status, DM, sex, PIR, marital status, and age. except for the stratifying variable.

We conducted stratified analyses to assess the eGDR‐depressive symptoms association's consistency in varying population subgroups. As depicted in Figure 3, utilization of eGDR as a continuous variable revealed no significant interactions across any of the subgroups, with the exception of the gender subgroup (p‐values for all other interactions exceeded 0.05). Given that the interaction is not significant, we have a belief that the associations exist in all subpopulations, and no evidence proved the existence of an interaction effect.

### 3.4 Sensitivity Analysis

We conducted sensitivity analyses (Table S3). Due to strong DM and TC correlations with eGDR, we excluded these variables from the covariate adjustment and stratified eGDR into quartiles to evaluate the strength of the correlation between depressive symptoms and eGDR. Results consistently showed an eGDR‐depressive symptoms association persisted after adjustments. In addition, this correlation remained significant after diabetic population exclusion (Table S4).

### 3.5 Assessing Predictive Capacity of TyG, HbA1C, HOMA‐IR, and eGDR for Depressive Symptoms

**FIGURE 4:**
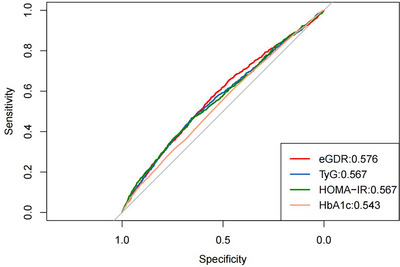
ROC curve analysis to predict depressive symptoms.

ROC curves were illustrated within Table 3 and Figure 4. In comparison to TyG, HOMA‐IR and HbA1C, eGDR demonstrates a superior predictive value (0.576 vs. 0.567 vs. 0.567 vs. 0.543, *P* < 0.05), with 7.446 optimal cut‐off value.

**TABLE 3:** Comparing ROC curves for IR indicators in predicting depressive symptoms.

Test	Best thresholds	Sensitivity	Specificity	AUC (95%CI)
eGDR	7.446	0.570	0.558	0.576 (0.558‐0.595)
TyG	8.801	0.466	0.657	0.567 (0.548‐0.586)
HOMA‐IR	3.276	0.468	0.651	0.567 (0.548‐0.586)
HbA1c	5.450	0.608	0.451	0.543 (0.524‐0.561)

Abbreviations: AUC, area under curve; eGDR, estimated glucose disposal rate; HbA1c, glycosylated hemoglobin A1C; HOMA‐IR, homeostatic model assessment for insulin resistance; TyG, triglyceride and glucose index.

### 3.6 Mediation Analysis

**FIGURE 5:**
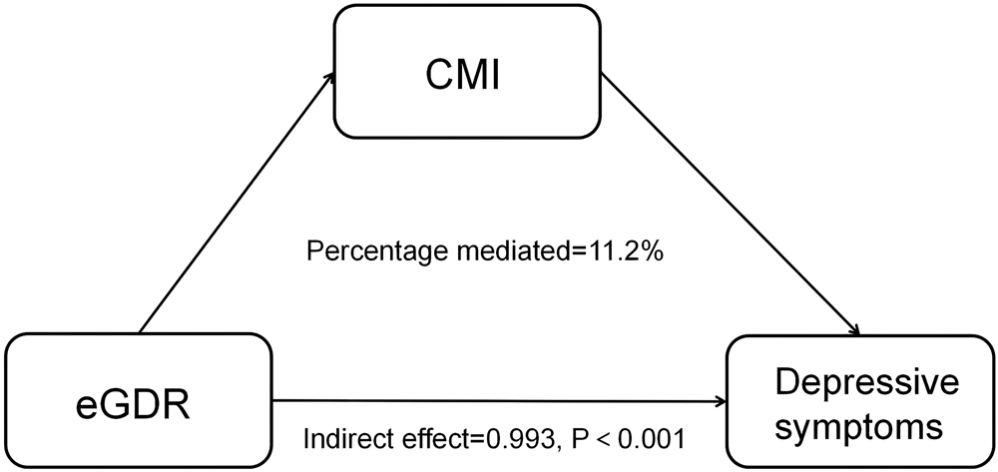
Proportion of the link between eGDR and depressive symptoms mediated by CMI.

Mediation effect was examined with eGDR as exposure, CMI as mediator, and depression as outcome. Results shown in Table 4 and Figure 5, revealed that CMI accounted for 11.2% (95% CI: 4.2‐32.7%) of the link between eGDR and depressive symptoms, with statistical significance (*p* = 0.004).

**TABLE 4:** Mediation analysis for relations between eGDR and depressive symptoms.

Characteristics	Effect	Total effect	Natural direct effect	Natural indirect effect	Percentage mediated (%)
CMI	OR (95% CI)	0.942 (0.908, 0.978)	0.948 (0.913, 0.985)	0.993 (0.990, 0.997)	11.2% (4.2%, 32.7%)
	*p*‐value	0.004	0.006	< 0.001	0.004

Abbreviations: CMI, cardiometabolic index; eGDR, estimated glucose disposal rate.

## 4 Discussion

Our observations demonstrate a significantly negative relation between elevated eGDR levels and depressive symptom risk, which remained robust even after comprehensive adjustments for potential confounders. Sensitivity and stratification analyses further reinforced the consistency and reliability of this relationship, which exhibited a linear pattern. ROC presented the superiority of eGDR in the prediction of depression, while mediation analysis further identified CMI as a partial mediator of this association.

Existing research has established eGDR as a reliable marker for assessing insulin resistance (Orchard et al. 2003). The negative impact of IR on depressive disorders has been well‐documented. For instance, a large South Korean cross‐sectional study (*N* = 165,443) observed that elevated IR was related to a 4% and 17% increased depression risk in young and non‐diabetic populations, aligning with multiple studies corroborating the IR‐depression link (Lee et al. 2017). Notably, the Netherlands Study of Depression and Anxiety identified significant connections between IR and chronic, treatment‐resistant depression, suggesting IR's potential role as a diagnostic marker for depressive disorders (Palladino et al. 2021). In our study, eGDR outperformed both the TyG index and HOMA‐IR in our investigation for predicting results. We hypothesize that eGDR's clinical utility stems from its three readily obtainable components, making it particularly suitable for large‐scale epidemiological studies requiring efficient identification of individuals at insulin resistance risk. Consistent with this perspective, eGDR has demonstrated clinical utility in large population studies for prognosticating mortality outcomes in type 2 diabetes mellitus (T2DM) patients and evaluating retinal morphology alterations in diabetes (Cui et al. 2023, Ryan and McLoughlin 2022).

Analysis reveals CMI's mediating role (11.2%) in the eGDR‐IR‐depression pathway, underscoring the importance of CMI surveillance in clinical practice. This aligns with prior research documenting significant CMI‐depressive symptom associations (Zhou et al. 2024). Therapeutic targeting of CMI may therefore represent a viable approach to mitigate depression risk among subjects with elevated eGDR levels. Nevertheless, the surveillance should not only focus on a singular index, but also on general cardiovascular and metabolic factors in clinical practice, in which hypertension, waist circumference, and HbA1c should also be under surveillance.

Although the precise mechanisms by which IR contributes to depression remain incompletely understood, several pathophysiological hypotheses have been proposed. First, increased IR accelerates atherosclerosis development and decreases in vascular dilatation function (Ramos Rego et al. 2021), which ultimately causes vascular depression (Czarny et al. 2018). Second, IR alters the regulation of several key neuroplastic mediators, which could disrupt neuronal plasticity and contribute to depression development (Tobe 2013). Third, low‐grade systemic inflammation, as frequently accompanied by chronic low‐grade inflammation, could adversely affect neuroplasticity, neurotransmitter metabolism, and neuroendocrine function, as well as promote apoptotic neuronal processes, all of which represent established pathways to depression (Penninx et al. 2024). Finally, oxidative stress, exacerbated by IR, leads to excessive free radical production that may cause neuronal damage and ultimately contribute to the development of depression (Yao et al. 2025).

Although ROC analysis demonstrated that the eGDR achieved a higher Area Under the Curve (AUC) compared to other established indices such as the TyG, HOMA‐IR, and HbA1c, it should be acknowledged that the absolute magnitude of eGDR's AUC remains relatively low. Consequently, further longitudinal investigations employing high‐quality study designs are imperative to robustly validate whether eGDR possesses superior predictive or diagnostic performance relative to these alternative indices, both within the general population and among other high‐risk subgroups.

The study presented several notable advantages. Firstly, the eGDR is well‐suited for extensive population‐based research, providing a practical approach to assessing IR across various patient populations. Secondly, the utilization of a nationally representative cohort strengthened the generalizability of our findings and enhanced the analytical rigor. Third, comprehensive potential confounding factors in the eGDR‐depressive symptoms link were carefully accounted for using multivariate regression approaches to ensure the robustness of the findings. Finally, the adoption of mediation analysis was employed to assess the potential pathway among lipid profiles, IR, and depression.

Several limitations should be acknowledged in this investigation. First, the cross‐sectional nature precludes causal inferences about the relationship between depression and eGDR. Second, as NHANES predominantly samples the U.S. population, result extrapolation to other ethnicities or regions requires caution. Third, depressive symptoms were assessed via single‐timepoint PHQ‐9 self‐reports referencing a two‐week period within the past year, which may not fully account for the dynamic nature of depressive symptoms. Furthermore, depressive symptoms exhibit considerable variability in severity, and these different levels may be related to the eGDR in distinct manners. Fourth, the dynamic changes in eGDR, which could offer a more robust reference value, were not captured in this investigation. Finally, despite accounting for numerous confounding factors, completely eliminating all potential confounders, such as participants' treatment status or insulin use among individuals with diabetes, remains a significant challenge.

## 5 Conclusions

After controlling for case complexity, a lower eGDR index was found to be linked to a higher likelihood of depressive symptoms within the general population, indicating that eGDR may serve as a simple yet effective index. Furthermore, for population‐based research, clinical approaches utilizing accessible and user‐friendly insulin resistance indexes, like eGDR, may be particularly practical.

## Author Contributions

**M. L**. and **C. C**. conceptualized the study, analyzed and interpreted the data, and drafted and revised the manuscript. **L. Y**. analyzed and interpreted the data, drafted and revised the manuscript, did the statistical analysis, and prepared all the figures. **S. W**., **W. H.,** and **X. Y**. did the interpretation of the data and revision of the manuscript. All authors contributed to the writing and revisions of the paper and approved the final version.

## Funding

Quanzhou City Science and Technology Program of China (No. 2024 NY026). Fujian Provincial Traditional Chinese Medicine Science and Technology Program(No.2025YBA007). The joint funds for the innovation of science and technology, Fujian province(Grant number: 2023Y9247).

## Ethics Statements

The protocol was approved by the Institutional Review Board of National Center for Health Statistics (protocol number: Protocol#98‐12, Protocol#2005‐06, Continuation of Protocol #2005‐06, protocol#2011–17 and Continuation of Protocol #2011–17).

## Conflicts of Interest

The authors declare no conflicts of interest.

## Supporting information




**Supplementary Material**: brb371247‐sup‐0001‐SuppMat.docx

## Data Availability

The original contributions presented in the study are included in the article/Supplementary Material. Further inquiries can be directed to the corresponding author.
