# Supplementary material for: Cardiometabolic Index as a Mediator in the Association Between Estimated Glucose Disposal Rate and Depressive Symptoms: A Population‐Based Study
Source: Brain Behav. 2026 Feb 5;16(2):e71247. doi: 10.1002/brb3.71247 (PMC12876042; doi:10.1002/brb3.71247)
Supplement: Supplementary file 1 — Supplementary Material: brb371247‐sup‐0001‐SuppMat.docx [file BRB3-16-e71247-s001.docx]

**Supplementary material**

**Table S1. The detail definition and classification of covariates.**

| **Variables** | **Definitions or Classification** |
| --- | --- |
| Sex | Male, Female. |
| Race | Non-Hispanic White, Non-Hispanic Black, Mexican American, Other Race. |
| Education attainment | High school or less, More than high school. |
| Marital status | Married or living with partner, Living alone. |
| PIR | Low (PIR ≤1), Middle (1< PIR < 4), and High (PIR ≥4). |
| Smoking status | Never smoking: <100 cigarettes in lifetime;  Former smoking: >100 cigarettes in life and smoke not at all now;  Now smoking: >100 cigarettes in lifetime. |
| Drinking status | Never: consumed fewer than 12 drinks in their lifetime;  Former: consumed at least 12 drinks in one year but did not drink in the last year or did not drink in the last year but had at least 12 drinks in their lifetime;  Heavy: consumed at least 3 drinks per day for females, at least 4 drinks per day for males, or engaged in binge drinking on 5 or more days per month;  Moderate: consumed at least 2 drinks per day for females, at least 3 drinks per day for males, or engaged in binge drinking on at least 2 days per month;  Mild : consumed at most 1 drink per day for females, at most 2 drinks per day for males. |
| Hypertension | An average systolic blood pressure (SBP) equal to or exceeding 140 mmHg;  An average diastolic blood pressure (DBP) equal to or exceeding 90 mmHg;  Self-reported hypertension;  Individuals taking prescribed anti-hypertensive medications. |
| DM | i) physician confirmation of diabetes diagnosis, ii) glycohemoglobin levels equal to or greater than 6.5%, iii) fasting glucose ≥ 7.0 mmol/L, iv) random blood glucose≥ 11.1 mmol/L, and v) documented use of DM medication. |
| CVD | The medical conditions section, identified by the variable name prefix MCQ, encompasses self- and proxy-reported personal interview data covering an extensive range of health conditions and medical history for both children and adults. This section includes inquiries such as ‘Has a doctor or other health professional ever informed you/SP that you/he/she… had congestive heart failure, coronary heart disease, angina (also called angina pectoris), heart attack (also called myocardial infarction), stroke, etc.?’ These questions, labeled as MCQ160B-F in the household questionnaires administered during home interviews, were utilized to identify participants with a history of CVD if they responded ‘yes’ to any of these questions. |
| Laboratory tests | The Specific method can be found in this webpage  ([https://wwwn.cdc.gov/nchs/nhanes/continuousnhanes/labmethods.aspx?BeginYear=200](https://wwwn.cdc.gov/nchs/nhanes/continuousnhanes/labmethods.aspx?BeginYear=2017)3) |

Abbreviations: PIR, family poverty income ratio; DM, diabetes mellitus; CVD, cardiovascular disease.

**Table S2. The risk factor of depression symptoms in NHANES 2003-2018^a^.**

| **Characteristic** | **Model 3**  **OR (95%CI), P value** |
| --- | --- |
| eGDR | 0.93 (0.87,0.99), 0.035 |
| **Age (years)** | 0.99 (0.99,1.00), 0.089 |
| **Sex (%)** |  |
| Male | Reference |
| Female | 1.88 (1.52,2.32), <0.0001 |
| **Race (%)** |  |
| Non-Hispanic White | Reference |
| Non-Hispanic Black | 0.97 (0.79,1.18), 0.746 |
| Mexican American | 0.84 (0.65,1.09), 0.195 |
| Other Race | 1.21 (0.93,1.57), 0.160 |
| **Educational Attainment (%)** |  |
| High school or less | Reference |
| More than high school | 0.85 (0.69,1.06), 0.149 |
| **Marital Status (%)** |  |
| Married or living with partner | Reference |
| Living alone | 1.66 (1.38,2.01), <0.0001 |
| **PIR (%)** |  |
| Low | Reference |
| Middle | 0.59 (0.48,0.72), <0.0001 |
| High | 0.36 (0.27,0.47), <0.0001 |
| **Smoking status (%)** |  |
| Never | Reference |
| Now | 2.74 (2.21,3.40), <0.0001 |
| Former | 1.43 (1.09,1.87), 0.009 |
| **Drinking status (%)** |  |
| Never | Reference |
| Mild | 0.91 (0.70,1.18), 0.472 |
| Moderate | 0.91 (0.69,1.21), 0.521 |
| Heavy | 1.12 (0.87,1.45), 0.369 |
| Former | 1.12 (0.80,1.56), 0.505 |
| **DM (%)** |  |
| No | Reference |
| Yes | 1.19 (0.94,1.50), 0.141 |
| **CVD (%)** |  |
| No | Reference |
| Yes | 1.70 (1.35,2.15), <0.0001 |
| **BMI** | 1.01 (0.99,1.03), 0.286 |
| **TC** | 1.00 (1.00,1.00), 0.157 |
| **eGDR** |  |
| **T1** | Reference |
| **T2** | 0.81 (0.64,1.02), 0.073 |
| **T3** | 0.68 (0.48,0.97), 0.031 |

**Table S3. The association between eGDR and depression after excluded DM and TC variables from the covariate adjustment.**

| **Characteristic** | **Adjusted OR (95%CI)** | **P-Vaule** |
| --- | --- | --- |
| eGDR | 0.92 (0.87,0.98) | 0.012 |
| **Categories** |  |  |
| Q1 | Reference |  |
| Q2 | 0.77 (0.59,0.99) | 0.045 |
| Q3 | 0.66 (0.47,0.93) | 0.018 |
| Q4 | 0.63 (0.42,0.95) | 0.029 |
| P for trend |  | 0.029 |

Q: quartiles; OR: odds ratio; CI: confidence interval.

Adjusted for age, sex, race, education attainment, marital status, BMI, PIR, smoking status, drinking status, CVD.

**Table S4. Sensitivity analysis between eGDR and depression after excluded DM patients.**

| **Characteristic** | **Model 1**  **OR (95%CI), P value** | **Model 2**  **OR (95%CI), P value** | **Model 3**  **OR (95%CI), P value** |
| --- | --- | --- | --- |
| eGDR (continuous) | 0.92 (0.89,0.95), <0.001 | 0.90 (0.86,0.94), <0.001 | 0.92 (0.88,0.96), <0.001 |
| eGDR (categorical) |  |  |  |
| T1 | Reference | Reference | Reference |
| T2 | 0.85 (0.67,1.07), 0.157 | 0.82 (0.65,1.05), 0.114 | 0.83 (0.64,1.06), 0.137 |
| T3 | 0.61 (0.48,0.77), <0.001 | 0.53 (0.40,0.71), <0.001 | 0.59 (0.44,0.79), <0.001 |
| P for trend | <0.001 | <0.001 | <0.001 |

eGDR, estimated glucose disposal rate; T, tertiles.

^a^Model 1: unadjusted; Model 2: adjusted for age, sex, race, educational attainment, and marital status; Model 3: adjusted for age, sex, race, education attainment, marital status, BMI, PIR, smoking status, drinking status,CVD, and TC.


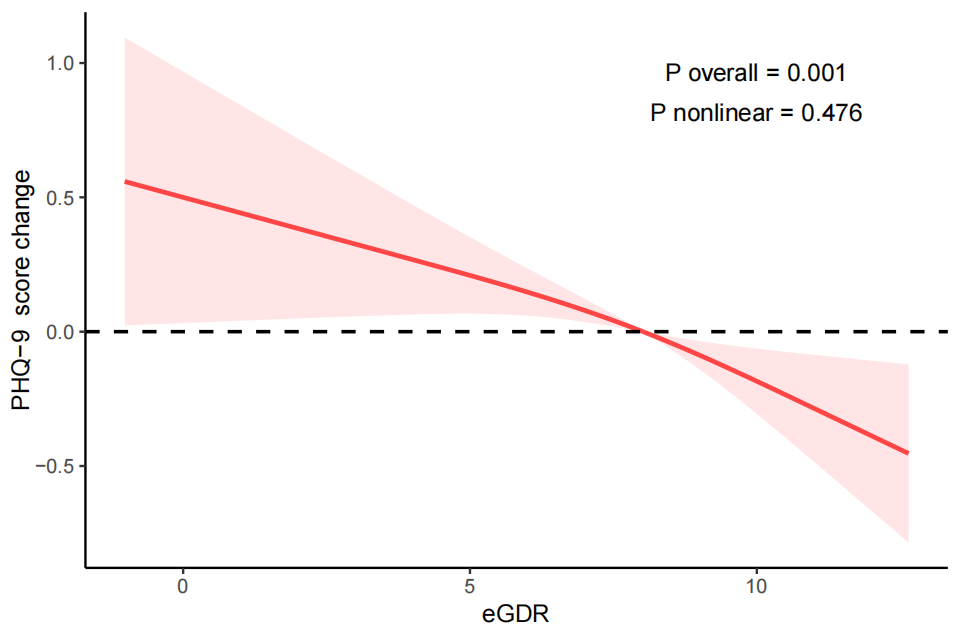


**Figure S1. Association between eGDR and PHQ-9 score. Adjustment factors included age, sex, race, education attainment, marital status, BMI, PIR, smoking status, drinking status, CVD, DM, and TC.**
